# Organ-Specific Distribution of Bioactive Compounds in *Valeriana microphylla* and Their Antioxidant, Antimicrobial, Haemolytic, and Nematicidal Activities

**DOI:** 10.3390/antiox15081031

**Published:** 2026-08-19

**Authors:** Elena Coyago-Cruz, Gabriela Méndez, Johana Zúñiga-Miranda, Alejandro Porras, Carlos Barba-Ostria, Jhennyfer Zambrano, Scarlet Recillo, Linda P. Guamán, Jorge Heredia-Moya, Blanca Naranjo, María Claudia Segovia-Salcedo

**Affiliations:** 1 Carrera de Ingeniería en Biotecnología, Universidad Politécnica Salesiana, Sede Quito, Campus El Girón, Av. 12 de Octubre N2422 y Wilson, Quito 170143, Ecuador; 2 Centro de Investigación Biomédica (CENBIO), Facultad de Ciencias de la Salud Eugenio Espejo, Universidad UTE, Quito 170527, Ecuador; 3 Departamento de Ciencias de la Vida y la Agricultura, Universidad de las Fuerzas Armadas ESPE, Quito 171103, Ecuador; 4 Escuela de Medicina, Colegio de Ciencias de la Salud Quito, Universidad San Francisco de Quito (USFQ), Quito 170901, Ecuador; 5 Instituto de Microbiología, Universidad San Francisco de Quito (USFQ), Quito 170901, Ecuador

**Keywords:** functional foods, bacteria, fungi, *Caenorhabditis elegans*, ATCC, multidrug-resistant microorganism

## Abstract

*Valeriana microphylla* is a medicinal species traditionally used in the Andean region. The study aimed to evaluate the bioactive compounds and the antioxidant, antimicrobial, haemolytic, and nematode locomotion-disrupting l activities in the flowers and leaves of *V. microphylla*. Minerals were quantified by atomic absorption spectrometry, whereas bioactive compounds were analysed by liquid chromatography. Antioxidant, antimicrobial and biological activities were determined by microplate spectrophotometry. Antimicrobial activity was tested against ATCC and multidrug-resistant microorganisms. Haemolytic activity was assessed by haemolysis of sheep erythrocytes, and nematode locomotion-disrupting activity by analysis of the locomotor behaviour of *Caenorhabditis elegans*. Potassium was the predominant mineral in leaves (2372.1 mg/100 g DW). In contrast, flowers contained the highest concentrations of citric acid (567.0 mg/100 g DW), total carotenoids (58.9 mg/100 g DW), and total phenolics (24,788.1 mg/100 g DW). Both extracts inhibited the growth of several ATCC bacterial strains, showing the lowest inhibitory concentrations against *Staphylococcus epidermidis*, *Edwardsiella tarda* and *Listeria monocytogenes*, but showed no activity against multidrug-resistant bacteria or against *Candida* species. Furthermore, both extracts exhibited haemolysis rates of less than 1.3% and affected the locomotion of *C. elegans* by eliminating the frequency of body bends and reducing locomotion speed. These findings provide an initial phytochemical and in vitro biological characterisation of *V. microphylla* and support further investigation of its bioactive constituents.

## 1. Introduction

The genus *Valeriana*, a member of the Caprifoliaceae family, comprises between 350 and 437 accepted species distributed worldwide [1]. It is particularly well represented in Andean ecosystems, where several species occur at altitudes above 2500 m above sea level [2]. The roots and rhizomes of different *Valeriana* species have traditionally been used in Andean, Asian, and European medicine, mainly for their sedative and anxiolytic properties and for the treatment of neurological disorders such as epilepsy [3,4,5]. This pharmacological relevance is supported by a complex phytochemical profile that includes iridoids (valepotriates), sesquiterpenoids (such as valerenic acid), lignans, flavonoids, and alkaloids—secondary metabolites known for their ability to modulate the central nervous system and protect tissues from cellular damage [6]. However, the available evidence is strongly influenced by species, geographical origin, plant organ, extraction solvent, and analytical method [7].

Furthermore, the abundance of phenolic compounds and flavonoids within the genus confers notable free-radical-scavenging capacity (often evaluated using DPPH and ABTS assays), positioning these extracts as natural alternatives to synthetic preservatives and as ingredients with the potential to reduce oxidative stress in the human body [7].

Most phytochemical and pharmacological studies have focused on a limited number of species, particularly *V. officinalis* and *V. jatamansi*. Extracts and isolated compounds from the species have been evaluated for antioxidant, neuroprotective, anti-inflammatory, and effective inhibition against foodborne pathogens and multidrug-resistant microorganisms, including *Staphylococcus aureus*, *Escherichia coli*, and *Candida albicans* [2].

Despite this potential, scientific research has focused disproportionately on fewer than 10% of the species in the genus, mainly those of Eurasian origin [7,8]. This knowledge gap is particularly evident in species like *Valeriana microphylla* Kunth, a plant native to the high Andean mountains whose traditional use to “cure the nerves” suggests latent bioactivity, yet it only possesses preliminary phytochemical reports regarding the presence of valepotriates (such as valtrate and didrovaltrate) and lignans like pinoresinol [1]. Compared with *V. officinalis* and *V. jatamansi*, its chemical composition and biological properties remain poorly characterised. Earlier phytochemical studies reported valepotriates, including valtrate and didrovaltrate, and lignans such as pinoresinol [1]. More recently, the essential oil obtained from the aerial parts of *V. microphylla* was characterised by GC-FID and GC-MS, revealing a chemically diverse volatile fraction and antioxidant activity, but no relevant acetylcholinesterase or butyrylcholinesterase inhibition [9]. This study provided important information on the volatile constituents of the species; however, essential oils differ chemically from hydroalcoholic extracts and do not represent the non-volatile compounds present in flowers and leaves. Consequently, findings obtained from Eurasian species or from roots and rhizomes cannot be directly extrapolated to the aerial organs of Andean *Valeriana* species.

Information remains limited regarding the organ-dependent distribution of minerals, organic acids, carotenoids, chlorophylls, and phenolic compounds in *V. microphylla*. Similarly, available studies have not provided an integrated comparative assessment of the antioxidant, antibacterial, antifungal, haemolytic, and nematode locomotion-disrupting effects of flower and leaf extracts. Addressing this gap is relevant because reproductive and photosynthetic organs may differ in their chemical composition and, consequently, in the biological responses of their extracts. Nevertheless, these relationships require direct evaluation and cannot be inferred from studies of other *Valeriana* species.

Although roots and rhizomes are traditionally regarded as the principal medicinal parts of *Valeriana* species, phytochemical and pharmacological research has consequently focused predominantly on these subterranean organs. In contrast, the flowers and leaves of *V. microphylla* remain poorly characterised. These aerial organs were selected to address this knowledge gap and to compare a reproductive tissue with a photosynthetic tissue, which may differ in mineral composition, primary metabolites, photosynthetic pigments, phenolic compounds, and other secondary metabolites.

Therefore, the aim of this study was to comparatively characterise the physicochemical properties, mineral composition, bioactive compounds, and antioxidant capacity of *V. microphylla* flowers and leaves and to evaluate the *in vitro* antibacterial, antifungal, and haemolytic activities of their hydroethanolic dry extracts, together with their effects on *Caenorhabditis elegans* locomotion. The novelty of the study lies in the integrated analysis of two previously under-investigated aerial organs of this Andean species using complementary chemical and biological assays. This exploratory characterisation provides a basis for future studies involving extract standardisation, untargeted metabolomics, bioassay-guided fractionation, mechanistic analysis, and *in vivo* validation.

## 2. Materials and Methods

### 2.1. Plant Material and Phytochemical Analysis of Valeriana microphylla Plants

**Figure 1:**
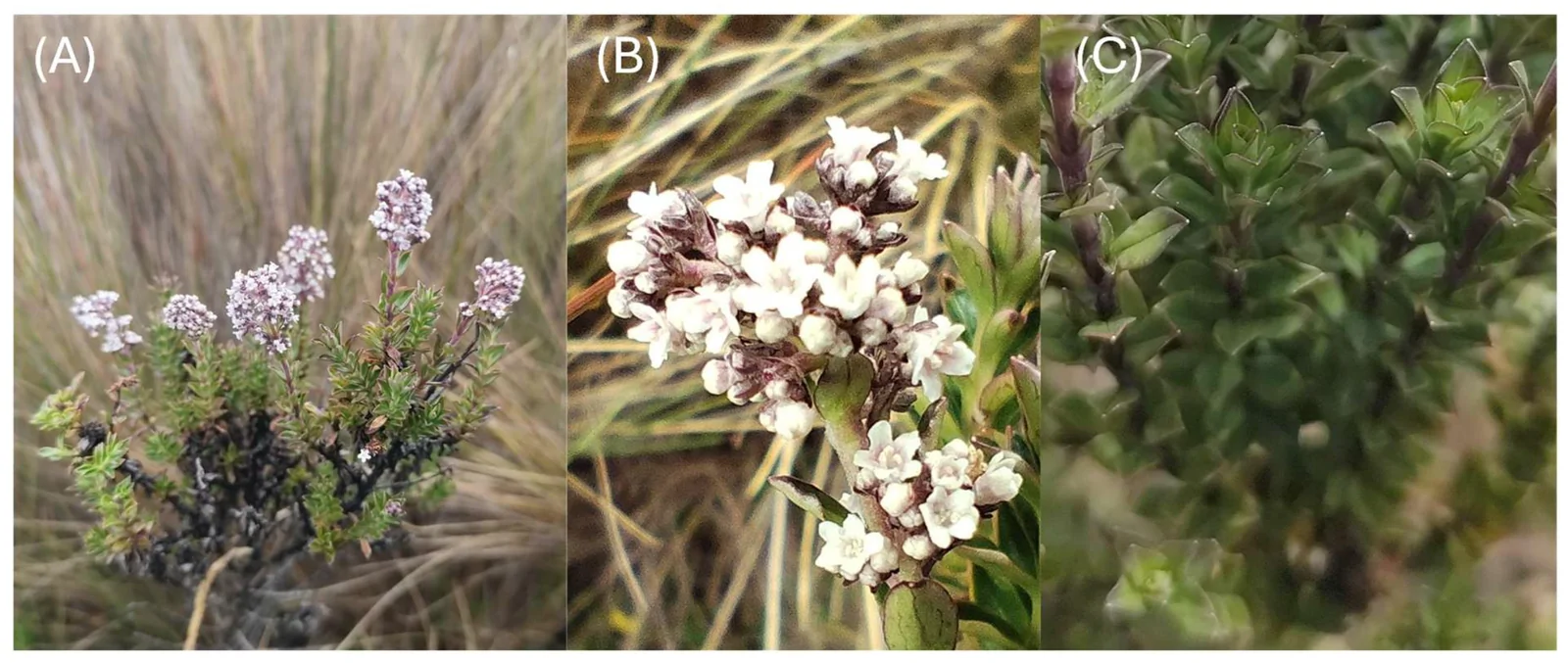
Photograph of ‘Mountain valerian’ (*Valeriana microphylla* Kunth). Note: (**A**) whole plant; (**B**) flowers; (**C**) leaves.

During March, flowers and leaves of *Valeriana microphylla* Kunth, commonly known as ‘Valeriana’ (Figure 1), were obtained from the Antisana moorland region located at 0°30′40.2″ S and 78°12′54.8″ W. Voucher specimens consisting of the entire plant were pressed and botanically authenticated at the Herbarium of the Universidad Politécnica Salesiana in Quito, Ecuador. Plant material collected at random from the same area, comprising twenty plants, was combined to produce two biological samples, one consisting of flowers and the other of leaves. Flowers and leaves were selected as the target organs because the study was designed to compare the chemical composition and biological activities of reproductive and photosynthetic aerial tissues. Roots and rhizomes were not included because the objective was specifically focused on the comparatively underinvestigated aerial organs of *V. microphylla*, rather than on the parts traditionally used for medicinal purposes. In addition, collecting aerial tissues allowed plant material to be obtained without removing the complete plant or its subterranean organs.

The floral and foliar tissues were then separated and divided into two sets. One set was used to determine physicochemical parameters, namely pH, soluble solids, titratable acidity, moisture, and ash content. The second set was freeze-dried using a Christ Alpha 1-4 LDplus lyophiliser (GmbH, Osterode am Harz, Germany). Finally, the dehydrated material was pulverised and maintained under storage conditions until further analyses were performed.

Phytochemical screening was performed to determine the presence or absence of broad metabolite classes, including acetogenins, alkaloids, anthraquinones, steroids, terpenoids, phenols, tannins, flavonoids, and saponins. This screening was intended as an exploratory analysis and was not used to estimate the concentration or relative abundance of these metabolite classes. The analysis was carried out in triplicate using qualitative colour-reaction, precipitation, or foam-formation tests, as appropriate for each metabolite class. Positive responses were determined visually according to the characteristic reaction criteria established for each test, in accordance with the methodology outlined by León-Fernández et al. [10]. Briefly, 20 mg of freeze-dried *V. microphylla* (flowers and leaves separately) were combined with 1 mL of deionised water. The mixture was homogenised and sonicated for 3 min in an FS60D ultrasonic bath (Intebiolab Inc., Orlando, FL, USA). The resulting supernatant was separated by centrifugation at 14,000 rpm and 4 °C for 5 min using an Eppendorf 5430 centrifuge (Eppendorf AG, Hamburg, Germany). The remaining pellet underwent two additional extraction cycles, each using 500 µL of deionised water. Afterwards, all recovered supernatants were pooled and employed for phytochemical evaluation.

Calcium, iron, potassium, and sodium contents were determined by atomic absorption spectrophotometry using a Varian Spectra A-55 instrument (Varian Inc., Palo Alto, CA, USA). Mineral extraction was performed in a Speedwave Xpert microwave digestion system (Berghof Products + Instruments GmbH, Eningen unter Achalm, Germany) equipped with Teflon vessels. The analysis was carried out in triplicate. Briefly, 40 mg of freeze-dried *V. microphylla* (flowers and leaves separately) was combined with 5 mL of 65% nitric acid and subjected to acid digestion at a temperature gradient set according to the equipment specifications. After digestion, the extracts were recovered and diluted to a final volume of 25 mL with deionised water. Calcium, iron, potassium, and sodium were analysed at wavelengths of 422.7, 372.0, 404.4, and 589.6 nm, respectively. Technical replicates were performed in triplicate, and the results were expressed as mg/100 g dry weight.

### 2.2. Analysis of Bioactive Compounds by Liquid Chromatography of Valeriana microphylla Plants

The evaluation of bioactive compounds included determining the levels of vitamin C, organic acids, carotenoids, chlorophylls and their derivatives, and phenolic compounds. Extractions were performed on 20 mg of freeze-dried *V. microphylla* powder (flowers and leaves separately) using microextraction after homogenisation and sonication in an FS60 ultrasonic bath (Fisher Scientific Inc., Waltham, MA, USA). The resulting supernatants were recovered by centrifugation at 14,000 rpm for 5 min at 4 °C in an Eppendorf 5430 centrifuge (Eppendorf AG, Hamburg, Germany) and filtered through a 0.45 µm PVDF filter. Chromatographic analyses were conducted using an RRLC 1200 system coupled to a DAD-UV-Vis detector (Agilent Technologies, Santa Clara, CA, USA). Specific chromatographic conditions, including wavelength, column, injection volume, and mobile phase composition, were adjusted according to each analysis. The mobile phase flow rate was fixed at 1 mL/min. Compound identification was performed with Lab ChemStation software (version 2.15.26). Quantification relied on calibration curves prepared with high-purity standards at 1 mg/mL. Compounds were identified by comparing their chromatographic retention times and UV-Vis absorption spectra with those of authentic reference standards analysed under the same chromatographic conditions. Quantification was performed using external calibration curves prepared individually from the corresponding reference standards. The concentrations were calculated from the integrated chromatographic peak areas and expressed as mg/100 g dry weight. Extractions were conducted in triplicate and chromatographic analyses in duplicate. Vitamin C extraction was carried out using 1.2 mL of 3% metaphosphoric acid and 200 µL of 0.2% *DL*-homocysteine. The solution was homogenised and agitated for 1 min prior to the addition of 600 µL of deionised water. Subsequently, the samples were centrifuged, and the supernatant was filtered before chromatographic analysis. Separation and detection were performed on an RRLC 1200 system at 244 nm using a ZORBAX Eclipse XDB-C18 column (1.8 µm, 4.6 mm × 50 mm) (Agilent Technologies, Santa Clara, CA, USA). The mobile phase consisted of 1.5% monobasic potassium phosphate and 1.8% *n*-acetyl-*n*,*n*,*n*-trimethylammonium bromide prepared in HPLC-grade methanol (90:10 *v*/*v*). A 20 µL injection volume was employed. Quantification was achieved using a calibration curve prepared with *L-*(+) ascorbic acid.

Organic acids were extracted using 1.5 mL of 0.02 N sulphuric acid supplemented with 0.05% metaphosphoric acid and 0.02% *DL*-homocysteine. The extract was homogenised and agitated for 3 min, then 500 µL of deionised water was added. The resulting solution was analysed in an RRLC 1200 chromatographic system at 210 nm using a YMC-Triart C18 column (3 µm, 4.6 mm × 150 mm) (YMC Europe GmbH, Dinslaken, Germany). The mobile phase consisted of 0.027% sulphuric acid. Samples were injected in a 20 µL volume. Quantification was performed through calibration curves prepared with malic, citric, and tartaric acids [11].

Carotenoids, chlorophylls, and their derivatives were extracted using 1.0 mL of a methanol:acetone:dichloromethane mixture (1:1:2). The suspension was homogenised and shaken for 1 min. After recovery of the supernatant, the remaining solid residue was repeatedly extracted with 500 µL of dichloromethane until the colour was completely removed. The combined extracts were evaporated to dryness at 30 °C using a Büchi R-100 rotary evaporator (Fisher Scientific, Hampton, NH, USA) [12]. The dried residue was reconstituted in ethyl acetate prior to chromatographic analysis. Separation and quantification were carried out on an RRLC 1200 system between 250 and 500 nm using a YMC C30 column (3 µm, 4.6 × 150 mm) (YMC Europe GmbH, Dinslaken, Germany). The mobile phases consisted of methanol (A), methyl tert-butyl ether (B), and water (C), employing the programmed gradient described in the method. The injection volume was 10 µL. The chromatograms were compared with α-carotene, β-carotene, β-cryptoxanthin, astaxanthin, trans-β-apo-8-carotenal, lycopene, lutein, violaxanthin, and zeaxanthin standards, and quantified using calibration curves for the major compounds identified. Chlorophylls and derivatives were quantified using pheophytin *a*, pheophytin *b*, chlorophyll *a*, and chlorophyll *b* standards.

Phenolic compounds were extracted using 1.0 mL of 80% methanol acidified with 0.1% HCl. The mixture was homogenised and shaken for 3 min, after which the supernatant was collected. The residual solid material was re-extracted twice with 500 µL portions of acidified methanol [13]. The combined extracts were analysed on an RRLC 1200 chromatographic system between 200 and 500 nm using a Zorbax Eclipse Plus C18 column (4.6 × 150 mm, 5 µm) (Agilent Technologies, Santa Clara, CA, USA). The mobile phases consisted of 0.01% aqueous formic acid (A) and acetonitrile (B), applying the following gradient: 0 min, 100% A; 5 min, 95% A + 5% B; 20 min, 50% A + 50% B; and 30 min for column washing and re-equilibration. The injection volume was 10 µL. The chromatograms were compared with *p*-hydroxybenzoic acid, 3-hydroxybenzoic acid, 2-methoxybenzoic acid, 3-methoxybenzoic acid, 2,5-dihydroxybenzoic acid, gallic acid, protocatechuic acid, vanillic acid, shikimic acid, syringic acid, ellagic acid, tannic acid, *p-*, *m-*, and *o*-coumaric acids, chlorogenic acid, caffeic acid, and ferulic acid. Additional standards included rutin, quercetin, myricetin, kaempferol, quercetin glucoside, chrysin, luteolin, catechin, naringenin, and naringin, and were quantified using calibration curves for the major compounds identified.

### 2.3. Antioxidant Activity of Valeriana microphylla Plants

Antioxidant activity was determined using the ABTS and DPPH assays in a BioTek H1 spectrophotometer equipped with a microplate reader (Agilent Scientific Instruments, Santa Clara, CA, USA). Extracts were prepared by mixing 20 mg of freeze-dried *V. microphylla* powder (flowers and leaves separately) with 2 mL of methanol, followed by homogenisation and sonication for 3 min in an FS60 ultrasonic bath (Fisher Scientific Inc., Waltham, MA, USA). The supernatant was recovered by centrifugation at 14,000 rpm and 4 °C for 5 min in an Eppendorf 5430 centrifuge (Eppendorf AG, Hamburg, Germany) and filtered through 0.45 µm PVDF membranes. Three independent extractions were prepared for each plant organ, and each extract was measured in duplicate. Quantification was performed using a Trolox calibration curve at 0.2 mg/mL, and results were expressed as mmol Trolox equivalents per 100 g dry weight.

The ABTS radical solution was prepared by mixing equal volumes of 7 mM ABTS and 2.45 mM potassium persulfate. The mixture was kept in darkness for 16 h and diluted with methanol until an absorbance of 0.7 at 734 nm was obtained. For analysis, 10 µL of extract was combined with 200 µL of ABTS solution, homogenised, and measured after 15 min of reaction.

The DPPH radical solution was prepared by dissolving 10 mg of DPPH in 50 mL of methanol and adjusting the absorbance to 0.9–1.1 at 515 nm. Antioxidant activity was quantified by mixing 20 µL of extract with 180 µL of DPPH solution, incubating for 40 min at room temperature in the dark, and measuring absorbance at 517 nm.

### 2.4. Antimicrobial Activity of Valeriana microphylla Plants

Hydroethanolic dry extracts of *V. microphylla* flowers and leaves were prepared separately using 50% aqueous ethanol. This solvent system was used to recover a broad range of polar and moderately polar constituents for exploratory biological testing. The extract was prepared by mixing 2 g of freeze-dried powder with 25 mL of 50% ethanol. The mixture was homogenised and agitated for 6 min prior to supernatant recovery. The remaining solid residue was extracted twice more with 25 mL portions of the ethanolic solution. The combined extracts were filtered through Whatman No. 1 filter paper (Cytiva, Maidstone, UK), concentrated using a rotary evaporator, and subsequently freeze-dried. Extraction yield was calculated as the mass of the recovered dry extract divided by the initial mass of freeze-dried plant material and expressed as a percentage. The yield of the flower extract was 32.2%, and that of the leaf extract was 31.4%. The dry extracts were stored in the dark and frozen until analysis. The resulting dry extract was employed for antimicrobial activity assays. Before each biological assay, the extracts were reconstituted in sterile distilled water to a stock concentration. Sterile distilled water was included as the corresponding vehicle control. All concentrations reported in the biological assays refer to the mass of dry extract per unit volume.

#### 2.4.1. ATCC Reference Strains

The minimum inhibitory concentration (MIC) of the hydroethanolic dry extract of *V. microphylla* (flowers and leaves separately) was assessed using the broth microdilution assay following CLSI recommendations, employing the bacterial strains *Staphylococcus aureus* ATCC 6538P, *Staphylococcus epidermidis* ATCC 12228, *Streptococcus mutans* ATCC 25175, *Escherichia coli* ATCC 8739, *Pseudomonas aeruginosa* ATCC 9027, *Pseudomonas fluorescens* ATCC 13525, *Pseudomonas putida* ATCC 31483, *E. faecalis* ATCC 49533, *K. pneumoniae* ATCC 10031, *Listeria monocytogenes* ATCC 19114, and *Edwardsiella tarda* ATCC 15947.

For antibacterial testing, the extract was first solubilised (400 mg in 2 mL of sterile distilled water) to obtain a concentrated preparation, from which a twofold dilution series was prepared directly in 96-well microplates previously dispensed with 100 µL of BHI broth, depending on the microorganism tested. The dilution series was generated by transferring equal volumes from one column to the next, ensuring a consistent decrease in concentration across the plate. The twofold dilution series covered final dry-extract concentrations from 150 mg/mL to 0.07 mg/mL. This range was selected based on previous studies, and the highest concentration corresponded to the maximum concentration that could be tested.

Microbial suspensions were adjusted to standardised densities (OD_600_ = 0.2 for bacteria), and 20 µL of each inoculum were added to the wells to achieve approximately 5 × 10^5^ CFU per reaction in a final volume of 120 µL. Incubation conditions varied by microorganism: *S. aureus*, *S. epidermidis*, *S. mutans*, *E. coli*, *P. aeruginosa*, *P. putida*, *E. faecalis*, *K. pneumoniae*, *L. monocytogenes* and *E. tarda* were incubated at 37 °C for 24 h, whereas *P. fluorescens* was incubated at 29 °C [14].

Each plate incorporated a sterility control, a growth control, and a reference inhibition control. Following incubation, metabolic activity was assessed by adding 20 µL of TTC reagent to each well and allowing a 1–2 h reaction period in the dark; the absence of red formazan indicated growth suppression. All determinations were performed in duplicate to ensure analytical consistency.

#### 2.4.2. Antifungal Activity

The antifungal activity of the ethanolic extracts of *V. microphylla* flowers and leaves was evaluated against four *Candida* species (*C. albicans*, *C. glabrata*, *C. krusei*, and *C. tropicalis*) using a modified broth microdilution growth-kinetics assay [15]. Each strain was grown overnight in YPD broth, and the cell density was adjusted to an OD_600_ of 0.1 using sterile distilled water. The inoculum was prepared by sequentially diluting the adjusted cultures in YPD to obtain the final cell suspension used in the assay.

The assay was performed in sterile 96-well microplates in a final volume of 200 µL per well using YPD medium. Hydroethanolic dry extracts from flowers or leaves were tested separately at final concentrations of 1250, 625, 312.5, 156.25, and 78.125 µg mL^−1^. Extract stock solutions were prepared in DMSO, and the assay was designed to maintain a constant final DMSO concentration of 2.5% across all extract-treated wells. Thus, across the concentration range tested, only the extract concentration varied, whereas the DMSO concentration remained unchanged. Appropriate growth controls, vehicle controls, and medium blanks were included in each plate.

Microplates were incubated at 35 °C in a Cytation 5 multimode reader (BioTek Instruments, Winooski, VT, USA), and *Candida* growth was monitored by measuring absorbance at 600 nm (OD_600_) every 30 min for 48 h. Antifungal activity was determined from the growth curves obtained for each treatment relative to the corresponding controls. Each condition was analysed using three replicates, and the complete experiment was performed independently three times.

#### 2.4.3. Multidrug-Resistant Isolates

The antibacterial activity of the hydroethanolic dry extract of *Valeriana microphylla* was assessed against seven multidrug-resistant bacteria—*Klebsiella pneumoniae*, *Escherichia coli*, *Salmonella enterica serovar Kentucky*, *Enterococcus faecalis*, *Staphylococcus epidermidis*, *Enterococcus faecium*, and *Pseudomonas aeruginosa*—using the microdilution method. These seven clinical multidrug-resistant isolates were provided by the National Health Institute of Ecuador (INSPI).

The bacterial inoculum was prepared in brain–heart infusion broth (BHI) to a final cell density of 5 × 10^5^ CFU/mL. Stock solutions of the tested extracts were prepared in DMSO at 25 mg/mL for *V. microphylla* flowers and leaves. Tested volumes were adjusted so the final concentration of the DMSO in each well was always 2.5% *v*/*v*. This concentration was previously shown not to affect bacterial growth [16]. As a control, bacterial cells were grown in the presence of 2.5% DMSO to rule out any potential growth-inhibitory effect. Additionally, nourseothricine (100 µg/mL) was used as a control for growth inhibition. Both BHI alone and supplemented with the extract were used as blanks.

Extract sensitivities were assessed using the microdilution method according to the Clinical and Laboratory Standards Institute (CLSI) guidelines [17], with the following modifications: first, 5 µL of each extract was added to 195 µL of bacterial suspension (5 × 10^5^ CFU/mL) to a final volume of 200 µL. The final concentrations in the wells were 125 µg/mL for *V. microphylla* flowers and leaves. The plates were then incubated at 37 °C for 20 h with constant shaking at 300 cpm (double orbital setting), and the OD_600_ was monitored every 30 min using a Cytation 5 multimode detection system (BioTek). The minimum inhibitory concentration (MIC) was determined by comparing the OD_600_ at 0 h with that after 24 h in samples exposed to the test suspension. These assays were performed at least in triplicate.

### 2.5. Haemolytic Activity of Valeriana microphylla Extracts

Briefly, 10 mL of defibrinated sheep blood was washed three times with 1× PBS, and the resulting erythrocytes were resuspended in 1× PBS to obtain a 1% suspension. This suspension was then mixed at a 1:1 ratio with *V. microphylla* extract at the indicated concentrations, with 10% Triton X-100 as a positive control and 2.5% DMSO as a negative control, in a 96-well polypropylene plate. After incubation at 37 °C for 1 h, the samples were centrifuged at 1700× *g* for 5 min. The supernatants were carefully transferred to a transparent flat-bottom 96-well plate, and absorbance was measured at 405 nm using a Cytation 5 multimode plate reader (BioTek). Each experiment was performed with three replicates and independently repeated three times. Haemolytic activity was assessed using the thresholds defined in the ASTM E2524-22 standard [18], ensuring alignment with contemporary hemocompatibility criteria.

The study utilised commercially available defibrinated sheep blood obtained from a quality-certified supplier. Since this is a standard biological reagent and did not involve the handling of live animals, the research was exempt from institutional animal ethics committee review. Standard biosafety protocols were followed throughout the study.

### 2.6. Nematode Locomotion-Disrupting Activity of Valeriana microphylla Extracts

The antinematicidal screening of hydroethanolic dry extracts obtained from *V. microphylla* flowers and leaves was evaluated using synchronised L1 larvae of the wild-type *Caenorhabditis elegans* strain N2. Synchronised larvae were obtained by hypochlorite treatment of gravid hermaphrodites, followed by overnight hatching in sterile M9 buffer at 20 °C. Freshly hatched L1 larvae, collected within 12–16 h after hatching, were used to minimise developmental variability among assays.

As a food source, *Escherichia coli* OP50 was grown overnight in LB medium at 37 °C with agitation. Cells were collected by centrifugation and inactivated by UV irradiation for 30 min. The UV-killed bacterial suspension was washed three times with sterile M9 buffer by centrifugation at 4400 rpm for 5 min, then resuspended in M9 buffer. The suspension was adjusted to an OD_600_ of 0.4 before use.

For each assay, a master mix was prepared in sterile M9 buffer containing synchronised L1 larvae, UV-inactivated OP50, and cholesterol. The larval density was adjusted to deliver approximately 50 L1 larvae per well. OP50 was included at an OD_600_ of 0.4 in the master mix, corresponding to a final OD_600_ of approximately 0.2 in the assay wells, and cholesterol was added to a final concentration of 5 µg mL^−1^. The suspension was gently but thoroughly mixed before dispensing to ensure a homogeneous distribution of larvae and bacteria.

Hydroethanolic dry extracts from flowers and leaves were prepared as stock solutions in DMSO and tested independently. Assays were carried out in sterile flat-bottom 96-well microplates in a final volume of 150 µL per well. Briefly, 75 µL of each extract dilution or control solution was first added to the wells, followed by 75 µL of the larva-containing master mix. Vehicle-treated wells were included as negative controls, whereas levamisole hydrochloride, at a final concentration of 50 mM, was used as a positive control for paralysis and mortality. When required, solutions were sterilised by filtration before use.

To reduce evaporation during incubation, sterile M9 buffer was added to the spaces between wells and to the peripheral reservoirs of the plates. Plates were incubated at 21 °C for up to 72 h under continuous gentle orbital agitation at 110 rpm, thereby keeping larvae in suspension and ensuring uniform exposure to the extracts.

Larval motility and viability were recorded at the start of the assay and every 24 h thereafter for up to 72 h. Motility was quantified by video recording followed by automated analysis using the wrMTrck plugin in Fiji/ImageJ (ImageJ 1.54p). Each treatment was analysed using at least 8 replicates, and the entire experiment was independently performed at least 3 times.

Data were expressed as mean ± SD or SEM, as indicated. Statistical differences between extract-treated larvae and their corresponding vehicle control were evaluated using the Kruskal–Wallis rank-based test, followed by an appropriate post hoc test. Extracts were considered to exhibit nematode locomotion-disrupting activity when they caused a statistically significant reduction in motility and/or viability relative to the vehicle control (*p* < 0.05).

### 2.7. Statistical Analysis

All variables evaluated across different plant parts were first subjected to descriptive statistical analysis. Results were expressed as mean ± standard deviation. The experimental unit and sample size for each assay are specified in the corresponding subsection. To determine statistically significant differences among the analysed plant parts, a one-way analysis of variance (ANOVA) was performed, followed by a post hoc test when appropriate. Differences were considered statistically significant at *p* < 0.05.

Additionally, principal component analysis (PCA) was conducted to explore the multivariate relationships among the samples and the evaluated variables. Since the variables were expressed in different units and measurement scales, the data were standardised using z-scores before analysis. PCA was performed using a correlation matrix and visualised with a biplot, in which samples were differentiated by plant part, while variables were projected as vectors.

Locomotion parameters were obtained from individual *C. elegans* tracks generated with WrmTrck/Fiji. For each recording, the values derived from all detected tracks were summarised as the per-video median, which was used as the experimental unit for statistical analysis. Thus, each video was treated as an independent biological replicate, yielding three observations per condition (n = 3 videos).

Differences among treatments were evaluated for mean locomotion speed (AvgSpeed, µm s^−1^), body-bend frequency (BBPS, bends s^−1^), normalised speed (BLPS, body lengths s^−1^), and cumulative track distance (µm). Because of the small sample size and the inability to reliably assess normality, global comparisons were performed using the Kruskal–Wallis rank-based test. Pairwise comparisons between the vehicle control (C−) and each *V. microphylla* extract were then carried out using two-sided Mann–Whitney U tests.

Effect size was estimated using the rank-biserial correlation coefficient, calculated as r = 1 − 2U/(n_1_ × n_2_), where U is the Mann–Whitney statistic and n_1_ and n_2_ denote the numbers of observations in the compared groups. Since, with n_1_ = n_2_ = 3, the lowest possible two-sided Mann–Whitney *p*-value is 0.100, pairwise *p*-values were not adjusted by Bonferroni correction. Instead, uncorrected *p*-values are presented together with effect sizes, allowing the magnitude and consistency of the observed effects to be evaluated directly. Trends were considered biologically meaningful when *p* ≤ 0.100 and r = −1.000, indicating complete rank separation between control and extract-treated groups. All statistical analyses were performed in Python 3.12 using the SciPy library.

## 3. Results

### 3.1. Phytochemical Analysis of Valeriana microphylla Plants

Table 1 summarises qualitative phytochemical screening, which showed organ-dependent differences in the metabolite classes detected in *V. microphylla*. Qualitative phytochemical screening showed differences in the metabolite classes detected in flowers and leaves. Steroids, phenols, tannins, and saponins were detected in flowers, whereas only saponins were detected in leaves under the assay conditions used.

Table 2 summarises the chemical analysis and mineral concentrations in the flowers and leaves of *V. microphylla*. Flowers and leaves showed similar soluble solids, titratable acidity, moisture, and ash contents, whereas leaf pH was slightly but significantly higher. Potassium was the predominant mineral in both organs and was approximately twice as high in leaves as in flowers. In contrast, flowers contained significantly more calcium, while sodium was higher in leaves. Iron was not detected in either organ under the analytical conditions used.

### 3.2. Bioactive Compounds by Liquid Chromatography

Table 3 summarises the concentrations of bioactive compounds in the flowers and leaves of *V. microphylla*. The chromatographic analysis revealed distinct bioactive-compound profiles in flowers and leaves. No significant differences between organs were observed in vitamin C and total organic acids. Citric acid predominated in both tissues, whereas flowers contained more citric and malic acids and leaves contained more tartaric acid. Among the carotenoids, those with the highest concentrations were lutein, 9-cis-antheraxanthin, violaxanthin, and β-carotene. Conversely, total chlorophylls and their derivatives were approximately twofold higher in leaves. In addition, among the phenolic compounds, gallic acid, vanillic acid, *m*-coumaric acid, 4-hydroxybenzoic acid, caffeic acid, and ferulic acid were present. Ferulic acid and naringin were the predominant phenolic compounds detected in both organs, and the total concentration of identified phenolics was higher in flowers than in leaves.

### 3.3. Antioxidant Activity

Table 4 summarises the antioxidant activity by the DPPH and ABTS methods, in the flowers and leaves of *V. microphylla*. Flowers and leaves showed comparable antioxidant capacity, with no significant differences between organs in either the DPPH or ABTS assay. For both tissues, the values obtained with DPPH were approximately twice those measured with ABTS.

### 3.4. Antimicrobial Activity

#### 3.4.1. ATCC Microorganisms

Table 5 shows the antimicrobial activity of *Valeriana* plant parts. Both extracts inhibited all ATCC bacterial strains within the concentration range evaluated, although susceptibility varied markedly among microorganisms. *S. epidermidis* was the most susceptible strain, with MIC values of 0.2 and 0.1 mg/mL for flower and leaf extracts, respectively. Low MIC values were also observed for *E. tarda* and *L. monocytogenes*. The largest organ-dependent difference was observed for *S. mutans*, which was more susceptible to the leaf extract, whereas *P. putida* showed the lowest susceptibility, particularly to the leaf extract.

#### 3.4.2. Antifungal Activity

No antifungal effect was detected for either the leaf or flower extracts against *Candida albicans*, *Candida tropicalis*, *Candida krusei*, or *Candida glabrata* under the concentrations evaluated.

#### 3.4.3. Multidrug-Resistant Microorganisms

We observed that the extracts exhibited no antibacterial activity against multidrug-resistant bacteria at the concentrations tested.

### 3.5. Haemolytic Activity of Flowers and Leaves of V. microphylla

Across the evaluated concentration range (7.81–125 µg/mL), both extracts exhibited low haemolytic activity, with HR values remaining below 1.3%.

For the flower extract, the highest HR was observed at 125 µg/mL, reaching 1.274 ± 0.014%. At lower concentrations (62.5–7.81 µg/mL), HR values decreased and remained close to the vehicle-control baseline. Similarly, the leaf extract exhibited its highest HR at 125 µg/mL, at 0.929 ± 0.003%. Upon dilution to 62.5, 31.25, 15.63, and 7.81 µg/mL, HR values decreased progressively to 0.861, 0.803, 0.617, and 0.115%, respectively.

At lower concentrations of the leaf extract, the mean optical absorbance values were slightly below those of the vehicle control. After conversion using the hemolysis calculation, these values corresponded to HR values below 1.0%.

### 3.6. Nematode Locomotion-Disrupting Activity

**Figure 2:**
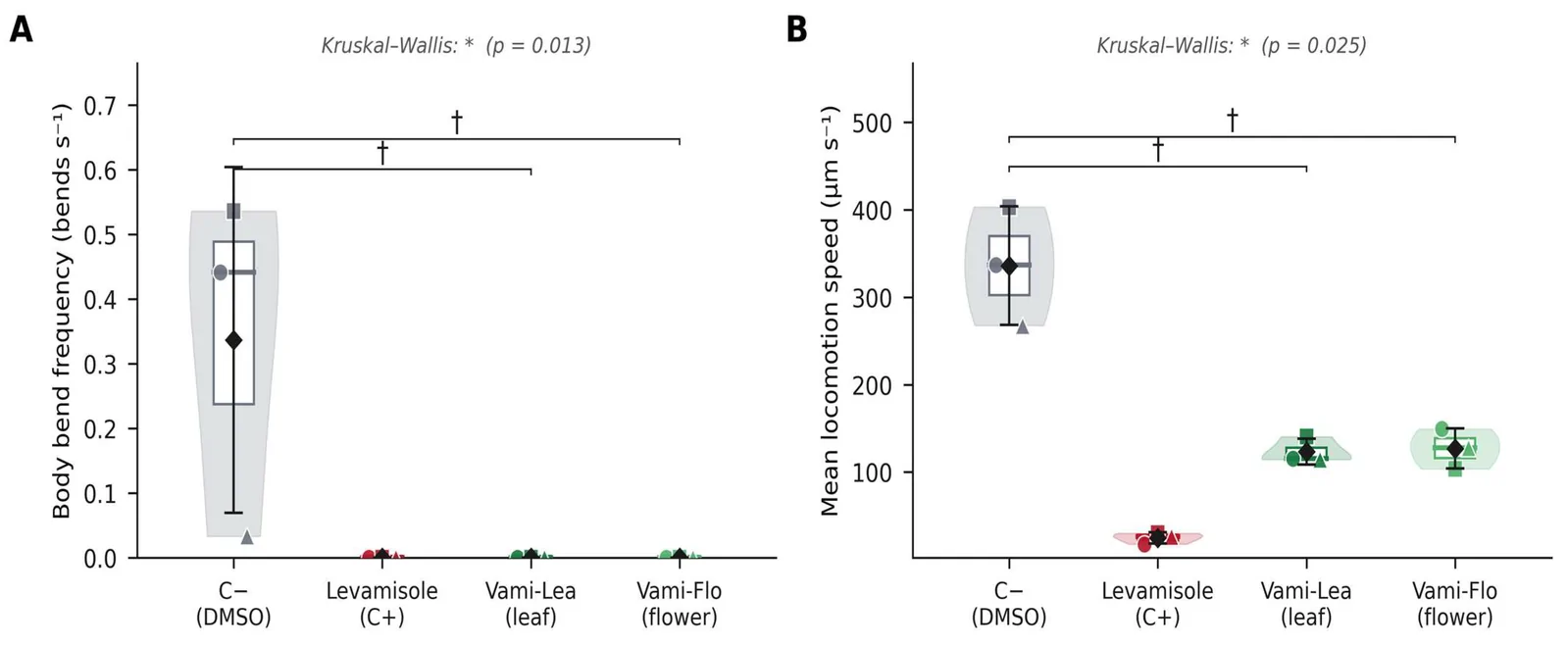
Effects of *Valeriana microphylla* hydroethanolic dry extracts on Caenorhabditis elegans locomotion. Body-bend frequency (**A**) and mean locomotion speed (**B**) were quantified in wild-type *C. elegans* N2 animals treated with *V. microphylla* leaf (Vami-Lea) or flower (Vami-Flo) ethanolic extracts. Vehicle-treated animals were used as a negative control (C−; DMSO), and levamisole as a positive control (C+; 20 µg mL^−1^). Three independent 20 s videos were analysed per condition. Each symbol represents the median value of all individual tracks detected in a single video, which is used as the unit of analysis. Circles, squares, and triangles denote Videos 1, 2, and 3, respectively. Violin plots show the distribution of per-video medians; white boxes indicate the interquartile range, horizontal lines the median, black diamonds the mean, and error bars ±1 SD. Colours indicate conditions: grey, C−; red, levamisole; dark green, Vami-Lea; light green, Vami-Flo. Brackets indicate pairwise comparisons with C−. †, uncorrected Mann–Whitney U *p* ≤ 0.10 with r = −1.000, denoting complete rank separation. Kruskal–Wallis H and *p* values are shown above each panel. Measurements were calibrated using 280 pixels = 1000 µm. BBPS is expressed as bends s^−1^ and AvgSpeed as µm s^−1^. Kruskal-Wallis test significance is indicated as * *p* < 0.05.

The principal locomotion parameters analysed were body-bend frequency, expressed as bends per second (BBPS; Figure 2A), and mean locomotion speed, expressed as µm s^−1^ (AvgSpeed; Figure 2B). Normalised locomotion speed, expressed as body lengths per second (BLPS), and cumulative track distance were included as supplementary parameters and are shown in Supplementary Figure S1A and S1B, respectively. Under vehicle control conditions (C−; DMSO), worms exhibited sustained, coordinated sinusoidal movement throughout the recording period. The median AvgSpeed values from the three control videos were 267.6, 337.0, and 403.2 µm s^−1^, yielding a mean ± SD of 335.9 ± 67.8 µm s^−1^. The corresponding BBPS values were 0.033, 0.441, and 0.536 bends s^−1^, with a mean of 0.337 ± 0.267 bends s^−1^. The proportion of tracks classified as active, defined as tracks with AvgSpeed > 10 µm s^−1^, was 94.4 ± 1.8%, and 39–47 individual tracks were detected per video.

As expected, levamisole markedly impaired locomotion. Treatment with this positive control resulted in a mean AvgSpeed of 24.6 ± 6.4 µm s^−1^, equivalent to 7.3% of the vehicle control. In addition, BBPS was reduced to 0.000 ± 0.000 bends s^−1^ in all three replicate videos, indicating complete suppression of detectable body bending (Figure 2A). The proportion of active tracks in levamisole-treated wells was 39.4 ± 29.7%, with 9–19 tracks detected per video.

Exposure to the leaf extract resulted in a pronounced reduction in locomotor activity relative to the vehicle control. The median AvgSpeed values obtained in the three replicate videos were 114.5, 115.3, and 140.4 µm s^−1^, yielding a mean of 123.4 ± 14.7 µm s^−1^. This value represents 36.7% of the control mean and corresponds to a 63.3% reduction in locomotion speed (Figure 2B). The effect was highly reproducible, as reflected by the lower coefficient of variation observed for leaf-treated animals (11.9%) compared with the vehicle control (20.2%). Strikingly, BBPS was 0.000 ± 0.000 bends s^−1^ in all three leaf replicate videos, corresponding to a complete loss of detectable body-bend activity and matching the value observed with levamisole (Figure 2A). However, the proportion of active tracks remained essentially unchanged relative to the vehicle control (94.3 ± 1.6% versus 94.4 ± 1.8%), indicating that leaf reduced coordinated undulatory movement without decreasing the proportion of animals classified as translationally active. Track detection was robust under this condition, with 104–183 individual tracks recorded per video.

A similar locomotor phenotype was observed in animals exposed to the flower extract. The three replicate videos yielded median AvgSpeed values of 103.7, 128.0, and 149.3 µm s^−1^, with a mean of 127.0 ± 22.8 µm s^−1^. Thus, flower-treated animals retained 37.8% of the control AvgSpeed and showed a 62.2% reduction relative to the vehicle control (Figure 2B). As observed for the leaf, body-bend frequency was completely abolished, with a BBPS value of 0.000 ± 0.000 bends s^−1^ in all replicate videos (Figure 2A). The proportion of active tracks remained comparable to that of the vehicle control (95.6 ± 2.6%), and 110–171 individual tracks were recorded per video.

The effects of leaves and flowers were quantitatively very similar. Mean AvgSpeed values differed by only 2.9 µm s^−1^, corresponding to less than 1% of the vehicle-control mean. Both extracts, however, allowed higher residual translational movement than levamisole, as indicated by AvgSpeed values of 123.4 ± 14.7 µm s^−1^ for leaves, 127.0 ± 22.8 µm s^−1^ for flowers, and 24.6 ± 6.4 µm s^−1^ for levamisole. In contrast, both extracts phenocopied levamisole with respect to body-bend frequency, since all Vami-treated videos yielded BBPS values of exactly 0.000 bends s^−1^.

Analysis of additional locomotion parameters supported the effects observed for AvgSpeed. Mean BLPS was reduced from 0.125 ± 0.033 body lengths s^−1^ in the vehicle control to 0.063 ± 0.011 body lengths s^−1^ in Vami-Ho-treated animals and 0.055 ± 0.010 body lengths s^−1^ in Vami-Flo-treated animals. These values correspond to reductions of 49.9% and 56.1%, respectively (Figure S1A). Consistently, cumulative track distance decreased from 182.5 ± 39.3 µm in the vehicle control to 85.6 ± 7.4 µm in Vami-Ho-treated animals and 80.0 ± 27.6 µm in Vami-Flo-treated animals, corresponding to reductions of 53.1% and 56.2%, respectively (Figure S1B). Levamisole produced the strongest reduction in these secondary parameters, with BLPS and cumulative distance values of 0.015 ± 0.008 body lengths s^−1^ and 5.1 ± 7.0 µm, respectively.

Kruskal–Wallis analysis revealed significant differences among treatments for all primary locomotion parameters: AvgSpeed (H = 9.359, *p* = 0.025), BBPS (H = 10.735, *p* = 0.013), BLPS (H = 9.667, *p* = 0.022), and cumulative track distance (H = 9.462, *p* = 0.024). Pairwise comparisons between the vehicle control and each *V. microphylla* extract using Mann–Whitney U tests yielded rank-biserial correlation coefficients of r = −1.000 for AvgSpeed, BBPS, BLPS, and cumulative distance for both leaves and flowers. Thus, for each parameter, all extract-treated replicate videos showed lower median values than all vehicle-control videos, indicating complete rank separation. The corresponding uncorrected Mann–Whitney *p*-values were 0.100 for AvgSpeed, BLPS, and cumulative distance, and 0.064 for BBPS, for both extracts. Given the sample size used here (n = 3 per condition), these values represent the strongest attainable two-sided Mann–Whitney evidence for a directional effect. No significant difference was detected in the proportion of active tracks between the vehicle control and either extract-treated condition (*p* > 0.400).

Together, these results indicate that the hydroethanolic dry extracts of *V. microphylla* leaves and flowers strongly impair *C. elegans* locomotor performance. Both extracts abolished detectable body-bend activity and substantially reduced translational speed, normalised speed, and cumulative displacement, while maintaining a proportion of active tracks comparable to that observed in vehicle-treated animals. This pattern suggests a robust disruption of coordinated sinusoidal locomotion rather than a simple loss of trackable animals.

### 3.7. Statistical Analysis

**Figure 3:**
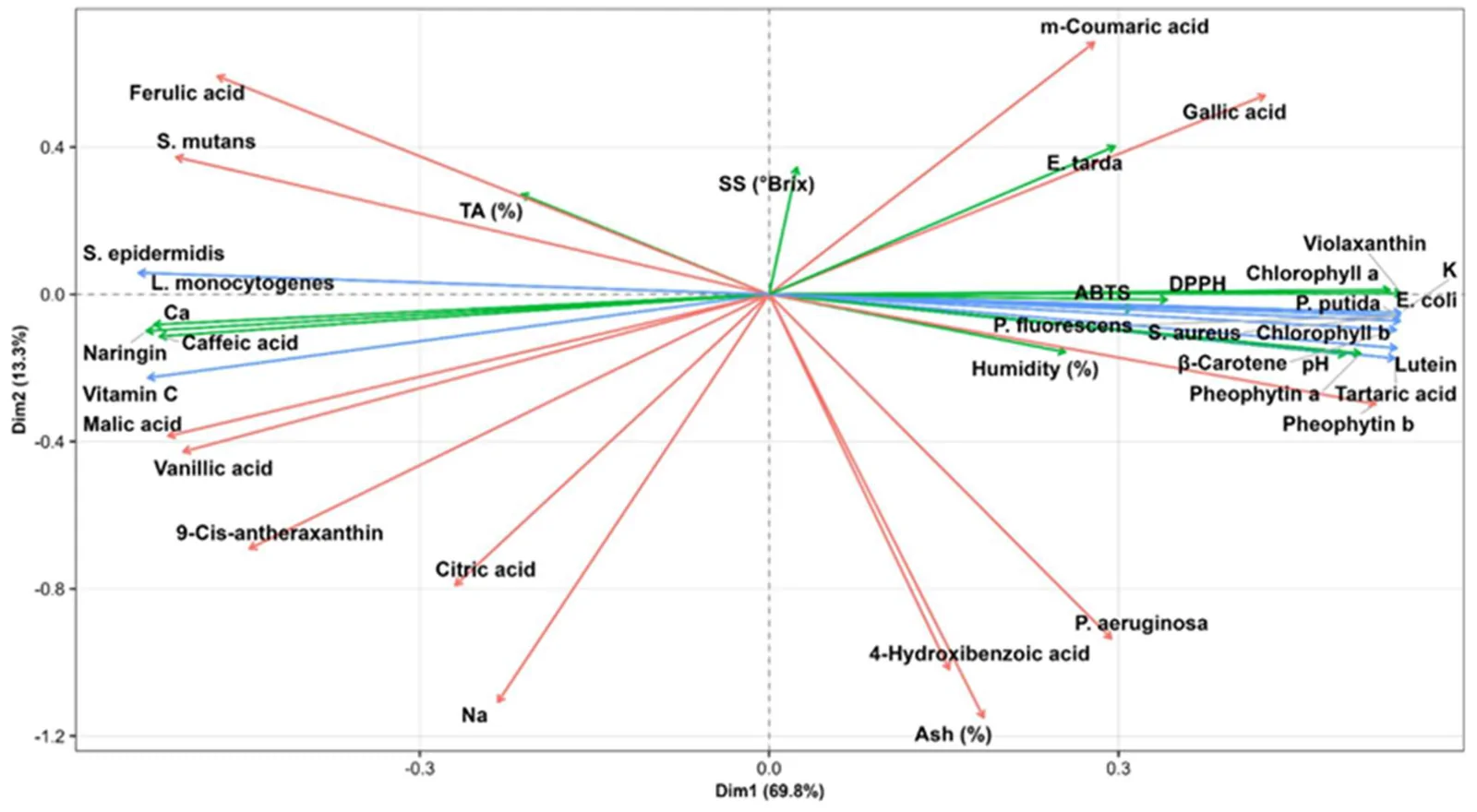
PCA biplot based on chemical composition and bioactive properties. Note: SS, soluble solids; TA, titratable acidity; ABTS, antioxidant activity by ABTS; DPPH, antioxidant activity by DPPH.

Figure 3 shows the principal component analysis for the variables under study. The first two principal components explained 83.1% of the total variance, with Dim1 accounting for 69.8% and Dim2 for 13.3%, indicating that the first two dimensions captured most of the dataset’s multivariate structure. Dim1 was the main axis of variation and separated variables with positive loadings, mainly related to pigment and carotenoid-associated parameters, including chlorophyll a, chlorophyll b, violaxanthin, β-carotene, lutein, pheophytins, potassium, pH, and antimicrobial responses against *E. coli*, *S. aureus*, *P. fluorescens and P. putida.* In contrast, negative Dim1 loadings were associated with several phenolic- and organic acid-related variables, including ferulic, caffeic, citric, vanillic, and malic acids, as well as naringin, calcium, vitamin C, and antimicrobial responses against *S. mutans*, *S. epidermidis*, and *L. monocytogenes*.

Dim2 represented a secondary source of variation, mainly separating variables located in the upper region of the biplot, such as *m*-coumaric acid, gallic acid, *C. albicans*, *E. tarda*, soluble solids, ferulic acid, and *S. mutans*, from variables positioned in the lower region, including ash content, 4-hydroxybenzoic acid, *P. aeruginosa*, sodium, citric acid, 9-cis-antheraxanthin, tartaric acid, and pheophytin b. Overall, the PCA suggests that the antioxidant and antimicrobial responses were associated with a complex multivariate profile involving phenolic acids, organic acids, pigments, carotenoids, and minerals, as other authors have pointed out [19].

## 4. Discussion

### 4.1. Phytochemical Screening

Qualitative phytochemical screening indicated organ-dependent differences in the metabolite classes detected in *V. microphylla*. Flowers showed positive reactions for steroids, phenols, tannins, and saponins, whereas leaves showed a positive reaction only for saponins. However, because these assays were qualitative, the results do not provide information on the concentrations or relative abundance of these metabolite classes. The organ-dependent distribution of secondary metabolites may be related to the different physiological and ecological functions of reproductive and photosynthetic tissues. Reproductive organs frequently accumulate specialised metabolites involved in defence against herbivores and pathogens, protection against ultraviolet radiation, and interactions with pollinators [20,21].

Furthermore, the physicochemical parameters of *V. microphylla* showed little statistical difference between flowers and leaves, suggesting a homogeneous composition in both organs. At the same time, the pH of the leaves was slightly higher than that of the flowers; this may be due to differences in the accumulation of secondary metabolites between photosynthetic and reproductive organs, since the leaves maintain intense photosynthetic activity and a more active primary metabolism, while the flowers allocate a greater proportion of carbon to the synthesis of specialised metabolites involved in attracting pollinators and defence mechanisms [22].

In terms of mineral composition, calcium was significantly higher in the flowers, while the leaves had a considerably higher concentration of potassium and sodium. The high concentration of calcium in the flowers can be explained by its structural role during reproductive development, as this element contributes to cell wall stabilisation, membrane integrity, and cellular signalling. Furthermore, calcium acts as an essential second messenger in the response to environmental stress, facilitating the regulation of multiple physiological processes related to the biosynthesis of specialised metabolites [21]. In turn, the high potassium content observed in the leaves is consistent with the physiological role of this macronutrient as the primary intracellular cation involved in osmotic regulation, stomatal opening, the transport of photoassimilates, and the activation of numerous enzymes involved in photosynthesis. The low concentration of sodium in plants promotes the simultaneous accumulation of potassium. The K/Na balance helps preserve enzymatic function and the synthesis of secondary metabolites responsible for the biological activities attributed to plants [23]. Furthermore, the absence of detectable iron in both organs may be due to low soil availability, environmental conditions, soil pH, and the species’ physiology [21].

### 4.2. Bioactive Compounds

No significant differences in vitamin C were observed between the flowers and leaves of *V. microphylla*, indicating that both tissues have a comparable capacity to accumulate ascorbic acid. Vitamin C is a key water-soluble antioxidant associated with the neutralisation of reactive oxygen species and with cellular protection against oxidative stress [24].

With regard to organic acids, citric acid was the predominant compound in both organs, with a higher concentration in flowers than in leaves, suggesting a greater accumulation of primary metabolism intermediates in floral tissue. Organic acids, such as citric and malic acids, are involved in plant energy metabolism and may contribute to acidity and chemical stability [25].

Furthermore, flowers exhibited significantly higher carotenoid concentrations. Carotenoids play essential roles in light capture, photoprotection, and dissipation of excess energy, and act as antioxidants against free radicals and singlet oxygen [26]. β-Carotene was the most abundant carotenoid in both flowers and leaves. This compound is recognised for its antioxidant activity and nutritional importance as a precursor to vitamin A [27].

Chlorophylls and their derivatives were significantly more abundant in leaves than in flowers. This result is expected, since leaves are the primary photosynthetic organs and contain pigments essential for light energy absorption [28].

In contrast to carotenoids and chlorophylls, total phenolic compounds were significantly higher in flowers than in leaves. This result indicates that flowers are the organ with the highest accumulation of phenolic metabolites in *V. microphylla*, which may be related to defence functions, protection against UV radiation, and interaction with pollinators [29].

Ferulic acid was the predominant phenolic compound in both organs, with a higher concentration in flowers. Ferulic acid is a hydroxycinnamic acid widely distributed in plants and has been described for its antioxidant, antimicrobial, anti-inflammatory, and neuroprotective activity [30]. Similarly, caffeic acid was higher in flowers, reinforcing the importance of floral tissue as an antioxidant. Hydroxycinnamic acids, including caffeic, ferulic, and coumaric acids, possess phenolic groups that can donate electrons or hydrogen, thereby contributing to the neutralisation of free radicals [29]. The concentrations of caffeic and ferulic acids should be interpreted in light of the influence of the extraction procedure, plant species, organ, population origin, and environmental and developmental conditions. Extraction conditions can substantially affect the recovery of bioactive compounds from *Valeriana* tissues [31]. Furthermore, qualitative and quantitative differences in phenolic composition have been reported between populations and phenological stages of *Valeriana carnosa* [32]. Because *V. microphylla* remains poorly characterised, species-specific quantitative data for these compounds are currently unavailable. The chemical profile observed in *V. microphylla* is consistent with the broad metabolic diversity described within the genus. LC-MS-based profiling of *V. jatamansi* has allowed the simultaneous detection and quantification of multiple bioactive constituents, illustrating the value of mass spectrometry-based approaches for characterising the chemical complexity of *Valeriana* extracts [1]. Therefore, future untargeted metabolomic analyses could complement the targeted chromatographic approach used in the present study.

### 4.3. Antioxidant Activity

The results of the antioxidant activity assays showed no significant differences between the flowers and leaves of *V. microphylla* in either the DPPH or ABTS assays. This finding suggests that both organs possess a similar capacity to neutralise free radicals, despite the previously observed differences in the composition of carotenoids, chlorophylls, and phenolic compounds. Other authors have demonstrated that the antioxidant activity of plant extracts depends on the combined effects of multiple bioactive metabolites [19], which may be additive or synergistic, compensating for differences in the individual concentrations of each chemical group. Furthermore, the differences between the assay methods may be due to the DPPH assay primarily evaluating the capacity for electron and hydrogen transfer against a lipophilic radical, whereas ABTS allows quantification of the antioxidant activity of both hydrophilic and lipophilic compounds [33].

### 4.4. Antimicrobial Activity

#### 4.4.1. ATCC Reference Strains

Flower and leaf extracts showed different inhibitory ranges among the ATCC strains.

Notably, the Minimum Inhibitory Concentration (MIC) values for *Staphylococcus epidermidis* were recorded as low as 0.1 mg/mL for leaves and 0.2 mg/mL for flowers. Similarly, significant inhibition was observed for *Edwardsiella tarda* (0.5–0.9 mg/mL) and *Listeria monocytogenes* (1.3–2.5 mg/mL), suggesting that the extracts contained constituents that may have contributed to the observed growth inhibition. In contrast, Gram-negative species such as *Pseudomonas aeruginosa* and *P. putida* exhibited high resistance, with MIC values up to 115.5 mg/mL, consistent with the intrinsic resistance mechanisms typically found in these genera against natural bioactive compounds.

In contrast to these findings, studies on pure compounds from *V. jatamansi*, such as the novel phenolic jatamanine, report significantly more potent MIC values of 4 mg/L (0.004 mg/mL) against *E. coli* and *C. albicans* [2]. Additionally, investigations into *V. officinalis* have shown that valerenic acid, a major sesquiterpenoid marker for the genus, does not possess significant antimicrobial activity on its own at doses up to 200 µg/mL [7]. This supports the hypothesis that the biological efficacy of the total extracts likely arises from synergistic interactions between phenolics characteristic of Andean species like *V. microphylla* [2,9].

#### 4.4.2. Antifungal Activity

The hydroethanolic dry extracts prepared from leaves and flowers of *V. microphylla* did not inhibit the growth of *Candida albicans*, *C. glabrata*, *C. krusei*, or *C. tropicalis* at any of the concentrations tested, from 1250 to 78.125 µg mL^−1^. This result was observed for both plant tissues and across the complete dilution series, indicating that these extracts lack detectable anti-*Candida* activity under the experimental conditions used. Thus, in contrast to their effect on *C. elegans* locomotion, the same preparations did not show broad antimicrobial activity against the fungal species evaluated.

Direct comparison with the literature is limited because antifungal studies in the genus *Valeriana* are scarce and have often focused on extracts or fractions that differ chemically from those tested here. Antimicrobial activity has been reported for *V. officinalis*, including against *Candida albicans*, but these data cannot be directly extrapolated to *V. microphylla* hydroethanolic dry extracts because species, plant organs, extraction methods, and chemical composition differ [34].

The lack of anti-*Candida* activity is also compatible with the phytochemical profile of the extracts. Several phenolic compounds detected in *V. microphylla*, including ferulic acid, caffeic acid, and related phenolic acids, have been reported to display antifungal activity against *Candida* when tested as isolated compounds or in defined preparations [35]. However, the activity of crude extracts cannot be inferred from the mere presence of these metabolites. The concentration of each compound within the extract, its chemical form, and possible interactions with other constituents may determine whether antifungal activity is observed. Accordingly, the negative result obtained here suggests that the phenolic composition of the leaf and flower extracts was not sufficient, in this assay format, to inhibit *Candida* growth.

Saponins were detected in both leaf and flower extracts and were proposed as candidate contributors to the locomotor phenotype observed in *C. elegans*. However, their presence does not necessarily predict antifungal activity. Studies with chemically defined C-27 steroidal saponins showed that activity against *Candida* spp. depends on the structure of the aglycone and on the number and arrangement of sugar residues; several saponins and all tested sapogenins were inactive at the highest concentration evaluated [36]. Since the saponins present in *V. microphylla* have not been structurally characterised, it cannot be determined whether they belong to active or inactive antifungal subclasses. The absence of anti-*Candida* activity is therefore not inconsistent with the detection of saponins in both extracts.

#### 4.4.3. Multidrug-Resistant Isolates

Hydroethanolic dry extracts of leaves and flowers of *V. microphylla* showed no antibacterial activity against the seven multidrug-resistant clinical strains evaluated (*K. pneumoniae*, *E. coli*, *S. enterica*, *E. faecalis*, *S. epidermidis*, *E. faecium and P. aeruginosa*) at 125 µg/mL, which contrasts with what has been reported for *V. jatamansi* and *V. triplinervis*, which inhibited similar pathogens at concentrations of 300–700 µg/mL with solvents of lower polarity [37,38]; this lack of activity may be explained by the insufficient concentration used, the use of 50% ethanol as an inefficient solvent to extract lipophilic compounds with greater antimicrobial potential [38] and the robust resistance mechanisms of MDR strains, which include efflux pumps, ESBL production and biofilm formation [39]. Future studies should evaluate extracts using nonpolar solvents, bioguided fractionation, and synergism with conventional antibiotics.

### 4.5. Haemolytic Activity

The hydroethanolic dry extracts prepared from leaves and flowers of *Valeriana microphylla* showed no measurable haemolytic activity against defibrinated sheep erythrocytes at the concentrations tested, from 78.125 to 1250 µg mL^−1^. This result is relevant because saponins were detected in both extracts, and several members of this compound class are known to disrupt erythrocyte membranes through interactions with cholesterol [40]. Thus, the absence of hemolysis indicates that the extracts were haemocompatible under the assay conditions used.

The result is also consistent with the structural dependence of saponin haemolytic activity. Haemolysis is not an intrinsic property of all saponins but depends on features such as the aglycone structure and the number and position of sugar chains. In *Chenopodium quinoa*, natural bidesmosidic saponins showed no haemolytic activity up to 500 µg mL^−1^, whereas hydrolysed derivatives were active at lower concentrations [41]. Since the saponins present in *V. microphylla* were not structurally characterised in this study, their haemolytic potential cannot be inferred solely from qualitative detection. The absence of erythrocyte lysis may reflect the presence of weakly haemolytic saponins, low concentrations of active saponins within the crude extracts, or interference by other extract constituents.

The phenolic compounds detected in both extracts, mainly ferulic acid, naringin, caffeic acid, and *m*-coumaric acid, are not expected to promote hemolysis. Consistently, leaf and flower extracts showed similar radical-scavenging capacity in the DPPH assay (5.9 ± 0.1 and 5.6 ± 0.3 mmol TE/100 g DW, respectively) and in the ABTS assay (2.9 ± 0.1 and 2.8 ± 0.2 mmol TE/100 g DW, respectively), with no significant difference between tissues (Table 4). Phenolic-rich extracts have been associated with antioxidant and antihaemolytic effects in erythrocyte-based assays [42]. Thus, the antioxidant capacity measured here is compatible with the absence of erythrocyte lysis. However, a protective effect on erythrocyte membranes cannot be inferred without specific antihemolysis assays. The similar antioxidant activity of leaf and flower extracts, despite their different total phenolic contents, also indicates that radical-scavenging capacity is not determined solely by total phenolic abundance.

### 4.6. Nematode Locomotion-Disrupting Activity

The present study shows that hydroethanolic dry extracts from leaves and flowers of *Valeriana microphylla* impair *Caenorhabditis elegans* locomotion. Both extracts abolished detectable body-bend activity, with BBPS values of 0.000 ± 0.000 bends s^−1^ in all replicate videos. Because body bending depends on the coordinated activity of the neuromuscular system, this phenotype indicates disruption of the motor program required for sinusoidal movement [43]. Levamisole, used here as a positive control, produced the same BBPS endpoint; however, its mechanism should be cited with a reference specific to levamisole-sensitive nicotinic acetylcholine receptors and body-wall muscle hypercontraction [44].

*C. elegans* is widely used in antinematicidal screening because its neuromuscular organisation, motor circuitry, and pharmacological sensitivity to anthelmintic drugs are well characterised and partly conserved among nematodes [1,9]. In this context, locomotion provides a functional readout of neuromuscular integrity. Body-bend frequency and translational speed are particularly informative, as coordinated sinusoidal movement depends on cholinergic and GABAergic motor circuits, which are targets of drugs such as levamisole and ivermectin [43]. Thus, inhibition of locomotion provides evidence of interference with nematode motor function. However, this endpoint alone does not distinguish reversible paralysis from lethality. Therefore, the complete suppression of BBPS observed here should be interpreted as evidence of locomotor impairment consistent with nematode locomotion-disrupting activity, whereas its nematostatic or nematicidal nature remains to be established by viability assays.

An important feature of the phenotype was the partial dissociation between body-bend frequency and translational movement. Although both extracts completely suppressed BBPS, mean locomotion speed was reduced to approximately 36–38% of the vehicle control, whereas levamisole reduced speed to approximately 7%. In addition, the proportion of tracks classified as active remained similar between extract-treated and vehicle-treated animals. Thus, the extracts did not simply eliminate trackable movement. Rather, they disrupted coordinated body undulation while allowing residual displacement, suggesting a mechanism distinct from generalised paralysis.

The phytochemical profile of *V. microphylla* provides a basis for interpreting this effect. Saponins were detected in both leaf and flower extracts and were the only major compound class shared by the two active preparations [7]. Because both extracts produced complete suppression of BBPS, saponins represent a plausible candidate class for the observed activity. This interpretation remains tentative, since the activity of the isolated saponin fraction was not tested. Nevertheless, saponin interactions with sterol-containing membranes could affect cells involved in neuromuscular coordination, including body-wall muscle cells.

The flower extract also contained condensed phenols and tannins. This is relevant because condensed tannins have been shown to act as anthelmintics in *C. elegans* by binding to the cuticle and increasing its rigidity, thereby affecting moulting and locomotion [45]. Therefore, the floral extract may act through more than one chemical class. However, because the leaf and flower extracts produced similar effects on BBPS and AvgSpeed, the present data do not establish whether the additional metabolites detected in flowers contribute to the phenotype or whether the shared saponin component is sufficient.

Phenolic compounds detected in both tissues may also contribute to the biological activity of the extracts. Ferulic acid, naringin, and caffeic acid were among the major compounds identified. Extracts of *Arceuthobium vaginatum* containing ferulic acid, naringin-related flavanones, and other phenolics inhibited *Haemonchus contortus* egg hatching [46]. Caffeic acid has also been reported to affect biological responses in *C. elegans*, including ATFS-1-dependent protection against pathogen infection [47]. These observations support the biological relevance of these compounds, but they do not demonstrate that they are responsible for the locomotor phenotype observed here. Bioassay-guided fractionation will be required to define the active constituents.

The activity observed for *V. microphylla* is consistent with the broader biological potential of the genus *Valeriana*. Species of this genus contain diverse secondary metabolites, including iridoids, sesquiterpenes, lignans, flavonoids, and phenylpropanoids [7]. Related Andean *Valeriana* species have been reported to contain alkaloids, flavonoids, tannins, sterols, triterpenoids, and saponins, and to display antimicrobial activity [38]. In addition, rhizome extracts of *V. wallichii* showed in vitro anthelmintic activity against *Pheretima posthuma*, causing paralysis and death in a concentration-dependent manner [48]. These reports support the view that *V. microphylla* belongs to a chemically and biologically relevant group, although its locomotion-disrupting activity has not been previously characterised.

Recent work on *V. microphylla* essential oil identified sesquiterpenes as the main volatile constituents and reported antioxidant activity, but no relevant inhibition of acetylcholinesterase or butyrylcholinesterase [9]. This observation argues against cholinesterase inhibition as the most likely explanation for the phenotype observed in the present study. The data are more consistent with effects on membrane, cuticular, or neuromuscular targets.

The use of *C. elegans* as a screening model is appropriate because its locomotion is well characterised and sensitive to compounds that affect neuromuscular function [43]. Plant-derived nematode locomotion-disrupting activity has also been evaluated in this organism; for example, dichloromethane and ethyl acetate fractions of *Annona crassiflora* leaf extract caused 98.13% and 89.66% larval immobility, respectively, and contained compounds such as quercetin and kaempferol [49]. In the present study, *V. microphylla* extracts completely suppressed body-bend activity, supporting their potential as a source of nematode-active metabolites.

On the other hand, the present study was restricted to flowers and leaves and therefore does not provide information on the chemical composition or biological activities of the roots and rhizomes, which are the organs most frequently used in traditional preparations of *Valeriana*. Consequently, the results should be interpreted as an organ-specific characterisation of the aerial tissues analysed and should not be extrapolated to the subterranean organs or to the whole plant. Thus, future studies should directly compare flowers, leaves, roots, and rhizomes collected from the same population and phenological stage to determine the extent of organ-dependent variation in the chemical and biological properties of *V. microphylla*.

## 5. Conclusions

*Valeriana microphylla* is an Andean species that exhibited a distinctive phytochemical profile for each organ studied. The flowers contained steroids, phenols, tannins and saponins, whilst the leaves contained only saponins. Furthermore, the flowers accumulated significantly higher concentrations of total phenolic compounds, particularly ferulic acid, whilst the leaves contained high concentrations of carotenoids, chlorophylls and potassium. In turn, both the flowers and the leaves exhibited similar antioxidant capacity in the DPPH and ABTS assays. Hydroethanolic dry extracts from both organs exhibited antibacterial activity against several ATCC reference strains, with the greatest inhibitory efficacy observed against *Streptococcus mutans*, *Staphylococcus epidermidis*, *Edwardsiella tarda* and *Listeria monocytogenes*. However, no inhibitory activity was detected against the *Candida* species evaluated or against multidrug-resistant bacteria. Furthermore, both the flower and leaf extracts exhibited haemolysis rates of less than 1.3%, indicating good in vitro biocompatibility under the experimental conditions used. With regard to nematode locomotion-disrupting activity, the extracts significantly altered the locomotor behaviour of *Caenorhabditis elegans*, completely suppressing the frequency of body bends and significantly reducing locomotor speed, whilst maintaining a proportion of active animals comparable to that of the control group. These results support the view that *V. microphylla* is a species with potential bioactive and biological effects.

## Figures and Tables

**Table 1:** Qualitative phytochemical screening of broad secondary metabolite classes in *Valeriana microphylla* flowers and leaves.

Metabolites	Flowers	Leaves
Steroids	+	−
Terpenoids	−	−
Phenols	+	−
Tannins	+	−
Alkaloids	−	−
Flavonoids	−	−
Anthraquinones	−	−
Saponins	+	+
Acetogenins	−	−

Note: +, detected; −, not detected under the assay conditions used. Qualitative phytochemical screening was performed using 3 independent extractions, with 3 technical determinations per extract.

**Table 2:** Average values from chemical analysis and mineral concentrations in the plant parts of *Valeriana*.

Parameters	Flowers	Leaves
pH	5.4 ± 0.1 ^b^	5.6 ± 0.0 ^a^
SS (°Brix)	0.3 ± 0.1 ^a^	0.3 ± 0.1 ^a^
TA (%)	0.1 ± 0.0 ^a^	0.1 ± 0.0 ^a^
Humidity (%)	64.4 ± 0.5 ^a^	64.7 ± 0.2 ^a^
Ash (%)	1.5 ± 0.1 ^a^	1.6 ± 0.2 ^a^
Mineral profile (mg/100 g DW)		
Ca	587.9 ± 15.0 ^a^	515.6 ± 16.4 ^b^
Fe	nd	nd
Na	25.6 ± 5.7 ^b^	31.4 ± 0.7 ^a^
K	1134.5 ± 4.0 ^b^	2372.1 ± 8.3 ^a^

Note: Mean values ± SD (n = 10). Different lowercase letters indicate significant differences between the aerial sections under study (*p* < 0.05, Tukey test). nd, undetectable; SS, soluble solids; TA, total titratable acidity; DW, dry weight.

**Table 3:** Average concentrations of bioactive compounds present in the plant parts of *Valeriana*.

Parameters	Flowers	Leaves
Vitamin C (mg/100 g DW)	13.5 ± 0.6 ^a^	13.5 ± 0.4 ^a^
Organic acid (mg/100 g DW)		
Citric acid	567.0 ± 10.3 ^a^	502.7 ± 46.1 ^b^
Malic acid	42.8 ± 3.2 ^a^	21.6 ± 7.4 ^b^
Tartaric acid	32.8 ± 5.4 ^b^	113.0 ± 5.8 ^a^
Total identified organic acid	642.7 ± 11.4 ^a^	637.4 ± 59.3 ^a^
Carotenoids (mg/100 g DW)		
Lutein	18.7 ± 0.6 ^b^	12.0 ± 0.2 ^a^
9-*cis*-antheraxanthin	0.6 ± 0.1 ^a^	0.4 ± 0.1 ^a^
Violaxanthin	2.6 ± 0.1 ^b^	1.4 ± 0.1 ^a^
β-Carotene	37.0 ± 1.9 ^a^	24.7 ± 0.5 ^b^
Total identified carotenoid	58.9 ± 2.7 ^a^	38.5 ± 0.3 ^b^
Chlorophylls and their derivatives (mg/100 g DW)		
Chlorophyll a	16.9 ± 4.6 ^b^	47.9 ± 0.2 ^a^
Chlorophyll b	33.5 ± 1.7 ^b^	59.1 ± 0.5 ^a^
Pheophytin a	9.4 ± 1.1 ^b^	12.2 ± 0.3 ^a^
Pheophytin b	nd	3.2 ± 0.7
Total chlorophyll	59.8 ± 1.8 ^b^	122.3 ± 0.2 ^a^
Phenolic compounds (mg/100 g DW)		
Gallic acid	2.6 ± 0.5 ^b^	4.2 ± 1.4 ^a^
Vanillic acid	103.2 ± 0.3 ^a^	90.9 ± 6.0 ^b^
*m*-Coumaric acid	1365.3 ± 28.7 ^b^	1712.8 ± 62.4 ^a^
4-Hydroxybenzoic acid	16.4 ± 1.8 ^a^	17.1 ± 1.2 ^a^
Caffeic acid	1842.2 ± 80.9 ^a^	1483.8 ± 95.6 ^b^
Naringin	5226.7 ± 24.6 ^a^	3570.5 ± 11.9 ^b^
Ferulic acid	16,231.6 ± 11.4 ^a^	9011.8 ± 33.3 ^b^
Total identified phenolics	24,788.1 ± 68.1 ^a^	15,891.1 ± 37.4 ^b^

Note: Mean values ± SD (n = 9). Different lowercase letters indicate significant differences between the aerial sections under study (*p* < 0.05, Tukey test)—nd, undetectable; DW, dry weight.

**Table 4:** Average antioxidant activity values of Valerian plant parts.

Parameters	Flowers	Leaves
DPPH (mmol TE/100 g DW)	5.6 ± 0.3 ^a^	5.9 ± 0.1 ^a^
ABTS (mmol TE/100 g DW)	2.8 ± 0.2 ^a^	2.9 ± 0.1 ^a^

Note: Mean values ± SD (n = 9). Different lowercase letters indicate significant differences between the aerial sections under study (*p* < 0.05, Tukey test). DW, dry weight.

**Table 5:** Minimum inhibitory concentration (MIC) of *Valeriana* plant parts.

Microorganisms	MIC (mg/mL)	
	Flowers	Leaves
*E. coli* ATCC 8739	30.5	62.5
*P. aeruginosa* ATCC 9027	60.9	62.5
*S. aureus* ATCC 6538P	15.2	15.6
*S. mutans* ATCC 25175	15.2	3.9
*P. fluorescens* ATCC 13525	29.0	57.7
*S. epidermidis* ATCC 12228	0.2	0.1
*P. putida* ATCC 31483	58.0	115.5
*E. tarda* ATCC 15947	0.5	0.9
*E. faecalis* ATCC 49533	10.2	10.2
*K. pneumoniae* ATCC 10031	40.6	40.6
*L. monocytogenes* ATCC 19114	2.5	1.3

## Data Availability

The original contributions presented in this study are included in the article. Further inquiries can be directed to the corresponding author.

## Supplementary Materials

The following supporting information can be downloaded at: https://www.mdpi.com/article/10.3390/antiox15081031/s1.
