# Supplementary material for: Organ-Specific Distribution of Bioactive Compounds in Valeriana microphylla and Their Antioxidant, Antimicrobial, Haemolytic, and Nematicidal Activities
Source: Antioxidants (Basel). 2026 Aug 19;15(8):1031. doi: 10.3390/antiox15081031 (PMC13510230; doi:10.3390/antiox15081031)
Supplement: Supplementary file 1 [file antioxidants-15-01031-s001.zip › antioxidants-4434300-supplementary.pdf]

Organ-specific distribution of bioactive compounds in *Valeriana microphila* and their antioxidant, antimicrobial, hemolytic, and nematicidal activities

Elena Coyago-Cruz[1*], Gabriela Méndez[1], Johana Zúñiga-Miranda[2], Alejandro Porras[3]; Carlos Barba-Ostria[4,5], Jhennyfer Zambrano[1], Scarlet Recillo[1], Linda P. Guaman[2], Jorge Heredia-Moya[2], Blanca Naranjo[3], M. Claudia Segovia-Salcedo[3]

SUPPLEMENTARY MATERIAL

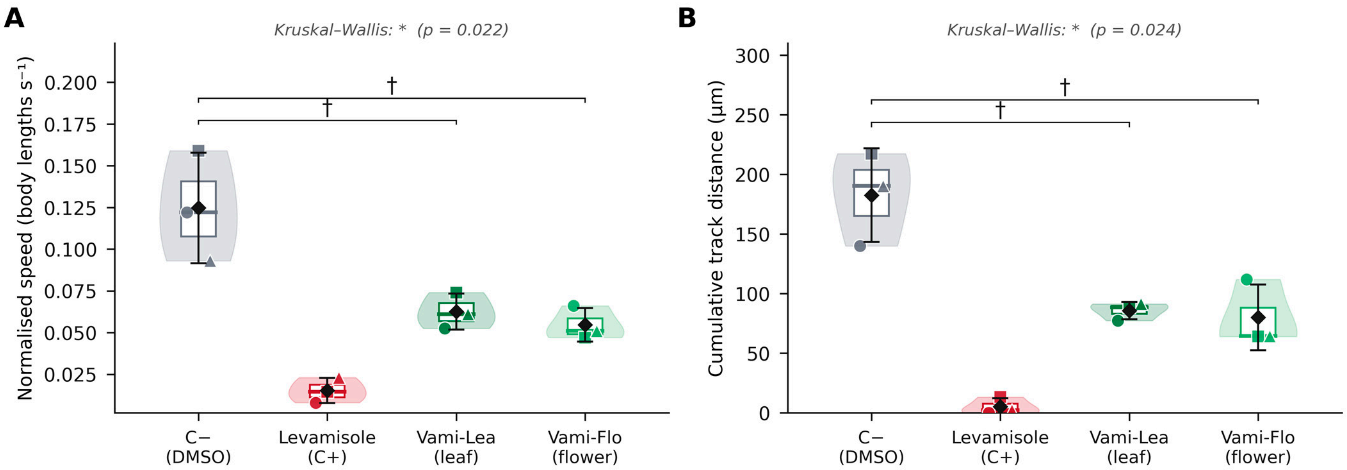

Supplementary Figure S1. Additional locomotion parameters of *Caenorhabditis elegans* treated with *Valeriana microphylla* ethanolic extracts. Normalized locomotion speed (A) and cumulative track distance (B) were measured in wild-type *C. elegans* N2 animals treated with *V. microphylla* leaf (Vami-Lea) or flower (Vami-Flo) ethanolic extracts. Vehicle-treated animals were used as the negative control (C−; DMSO), and levamisole-treated animals as the positive control (C+; 20 µg mL^{-1}). Data were acquired and processed as described for Figure 2. Each condition included three independent 20-s videos; each point represents the median value of all tracks detected in one video. Circles, squares, and triangles indicate Videos 1, 2, and 3, respectively. Violin plots show the distribution of per-video medians; white boxes indicate the interquartile range, horizontal lines the median, black diamonds the mean, and error bars ±1 SD. Colors indicate conditions: grey, C−; red, C+; dark green, Vami-Lea; light green, Vami-Flo. Brackets indicate pairwise comparisons with C−. †, uncorrected Mann–Whitney U $p \leq 0.10$ with rank-biserial correlation $r = -1.000$. Kruskal–Wallis H and p values are shown above each panel. Measurements were calibrated using 280 pixels = 1,000 µm. BLPS is expressed as body lengths s^{-1}. Cumulative track distance is expressed in µm and corresponds to the total path length recorded for each worm track.
